# Human brown fat and metabolic disease: a heated debate

**DOI:** 10.1172/JCI176678

**Published:** 2023-12-01

**Authors:** Rana K. Gupta

**Affiliations:** Department of Medicine, Division of Endocrinology, and Duke Molecular Physiology Institute, Duke University Medical Center, Durham, North Carolina, USA.

Mammals maintain thermoregulation in the face of cold environmental temperatures by activating bioenergetic mechanisms that increase heat production. Included in this response is both shivering (rapid contraction and relaxation of skeletal muscles) and nonshivering thermogenesis. Nonshivering thermogenesis involves the activation of energy-expending brown adipose tissue (BAT), a mitochondria-rich fat tissue that utilizes multiple futile energy-cycling mechanisms to produce heat. Moreover, in response to cold, white adipose tissue (WAT) undergoes a pronounced thermogenic remodeling in which this energy-storing fat tissue adopts a BAT-like phenotype with the emergence of thermogenic fat cells called beige adipocytes. From both a basic science and translational perspective, the study of adipose thermogenesis and its relationship to systemic energy balance remains of great interest. The thermogenic response of adipose tissue exemplifies the remarkable plasticity of this tissue at multiple levels. The study of adipose thermogenesis offers a lens through which several aspects of mitochondrial function, nutrient oxidation, cell differentiation, and tissue remodeling can be explored. The potential for adipocyte thermogenesis to increase metabolic rate makes brown fat cells, in principle, an attractive therapeutic target to treat obesity and cardiometabolic diseases.

## 2008 to 2009 marked a turning point

In 2008, Seale, et al. reported the surprising finding that murine brown adipocytes emerging during development emanate from *Myf5*^+^ cells of the skeletal muscle lineage (1). The idea that brown adipocytes and skeletal muscle share a common ancestry challenged the longstanding concept that brown and white adipocytes arise from a common adipoblast. Equally surprising was the finding that thermogenic adipocytes that emerge within WAT depots upon cold exposure do not emanate from a *Myf5*^+^ skeletal muscle lineage, providing evidence of the existence of two developmentally distinct types of thermogenic adipocytes (classical brown and beige adipocytes, respectively). Seale, et al. also discovered that the transcriptional coregulator PRDM16 acts as a brown fat lineage determination factor controlling the skeletal muscle versus brown adipocyte lineage decision (1). Contemporaneously, Tseng, et al. identified bone morphogenic signaling protein 7 (BMP7) signaling as a critical orchestrator of the brown adipocyte differentiation program during development (2). From my point of view at the time (as a trainee in 2008), these breakthrough studies reinvigorated the field of adipocyte development as they opened the doors to several new research directions and helped imagine possibilities for how energy-burning brown adipose tissue could be engineered. The importance of these studies was amplified in 2009 by contemporaneous landmark studies from multiple labs reporting the existence of cold-activated BAT depots in adult humans, revealed using ^18^F- fluoro-deoxyglucose (^18^F-FDG) uptake with PET and CT imaging (3–5). Prior to this work, the prevailing view was that BAT depots observed in infancy were lost in adulthood. This, so-to-speak, rediscovery of human BAT raised the possibility that BAT plays an important role in maintaining energy balance and nutrient homeostasis in adulthood and that this tissue may indeed be a viable target of therapeutic inventions designed to combat metabolic disease.

**Figure 1:**
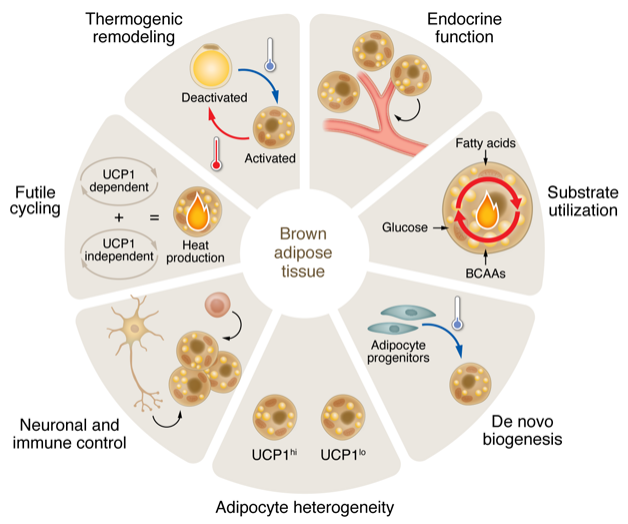
Several important areas of investigation in the field of BAT biology relate to cardiometabolic disease. Rapidly emerging functions and areas of study in BAT biology incite multiple tissues and processes. For example, neuronal and immune signaling exert a regulatory effect on brown fat cells, cold temperatures induce de novo brown adipocyte differentiation, and substrates beyond glucose can fuel thermogenesis. Brown fat cells also exert control over endocrine functioning and show heterogeneity with specific metabolic activity. Moreover, WAT can respond to cold temperatures by remodeling to a BAT-like phenotype. While UCP1 generates heat by disruption of the mitochondrial inner membrane electrochemical gradient, UCP1-independent mechanisms that fueling adipocyte thermogenesis also exist. Understanding the interplay between these systems may lead to strategies for harnessing the power of BAT in the treatment for human metabolic disease.

Over the next 15 years, our knowledge of brown and beige adipocyte biology rapidly expanded. New concepts that fundamentally change the way we think about BAT are continuously emerging. Today, some of the exciting areas of investigation include the endocrine function of brown fat cells, mechanisms controlling de novo brown adipocyte differentiation, fuel preference for thermogenesis, and the neuronal and immune cell signals that modulate tissue plasticity (Figure 1). For example, the longstanding view was that UCP1-mediated disruption of the mitochondrial inner membrane electrochemical gradient represented the sole thermogenic mechanism within brown adipocytes. Now it is clear that UCP1-independent mechanisms fueling adipocyte thermogenesis exist, including futile calcium cycling, lipid-cycling, and creatine metabolism (6). The identification of such mechanisms is exciting as it potentially opens additional avenues for intervention. In addition, single-cell transcriptomics has unveiled the cellular heterogeneity of BAT, including the presence of multiple progenitor cell populations and subsets of adipocytes with seemingly distinct metabolic activity (7). Moreover, single-cell sequencing has helped unveil important cell-cell interactions driving the formation and activation of beige and brown adipocytes within WAT depots (8). Rodent models of enhanced brown and beige fat function continue to highlight the beneficial impact of BAT activation and thermogenic WAT remodeling on energy expenditure, glucose homeostasis, and cardiovascular health. The latter can occur via its role as an endocrine organ or as a metabolic sink. Importantly, both brown and beige fat cells can impact nutrient homeostasis even independently of its impact on energy expenditure. This point is critical, as it dispels the notion that BAT is merely a heat generator.

## A 2023 debate focuses on human BAT

In parallel to the studies of rodent thermogenic adipose tissue, efforts to understand the importance of BAT in humans have continued. Notably, Becher et al. determined that the presence of BAT in adult humans is linked to the incidence of cardiometabolic disease (9). Individuals with detectable BAT had lower prevalence of type 2 diabetes, dyslipidemia, congestive heart failure, and hypertension, across different ranges of BMI. This observation is exciting as it supports the hypothesis that BAT can regulate cardiometabolic health, even independent of its potential effect on body weight. Nevertheless, correlation, of course, does not equal causation. As such, the physiological importance of BAT in adult humans and the potential contribution of BAT dysfunction to the development of metabolic disease has remained a topic of great discussion. In June of 2023, organizers of the annual meeting of the Endocrine Society (ENDO 2023) staged a live debate, where leading investigators in the study of human BAT offered their perspectives on the burning question of whether human BAT is a viable target for treatment of cardiometabolic disease. In this issue of the *JCI*, these same investigators expand on the discussion, highlighting the challenges (Carpentier and Blondin) and promise (Cypess) of human BAT as a therapeutic target (10, 11).

Carpentier and Blondin question the physiological importance of human BAT during acute cold exposure, as the amount of BAT is relatively small in comparison with rodents and cold exposure invokes a multi-organ response (e.g., shivering thermogenesis) in which the contribution of BAT may be minor. Moreover, they raised concerns as to whether ^18^F-FDG uptake truly reflects BAT thermogenesis, particularly in older or diabetic subjects, arguing that glucose uptake and metabolism may not be directly coupled to thermogenesis. As such, they question whether there is sufficient evidence that BAT dysfunction contributes to development of metabolic disease. Substrate preference for BAT thermogenesis has been a topic of discussion. The development of improved stable isotope tracing techniques is now enabling investigators to elegantly trace nutrient fate and identify critical fuels of thermogenesis in adipocytes (12, 13). Emanating from these emerging studies is an appreciation of the flexibility of brown adipocytes with respect to fuel selection and the importance of nutritional state (fed versus fasted) in determining the preferred fuel choice (10). Multiple factors may influence substrate utilization in BAT. For example, mice display a diurnal rhythm of BAT thermogenesis, including rhythmic futile creatine cycling, which is highest during the start of the active/dark period (14). Diurnal BAT activity may be an important factor to consider when studying the therapeutic potential of promoting BAT activity. Other additional experimental factors may need to be considered in the study of BAT thermogenic activity, such as the duration of cold exposure.

Cypess remains optimistic about the potential of pharmacological BAT activation to drive improvements in glucose and lipid homeostasis. Treatment of individuals with the FDA-approved β3-adrenergic receptor agonist, mirabegron, leads to an estimated doubling of BAT mass, increased energy expenditure, increased insulin sensitivity and insulin secretion, and elevated HDL (15). Increasing energy expenditure did not ultimately impact fat mass and body weight, perhaps due compensatory changes in food intake. Cypess acknowledges that activating BAT as a therapeutic for obesity is likely a big challenge; however, BAT activation through this mechanism may help in the treatment of glucose and lipid disorders. The choice of utilizing β3-adrenergic receptor agonists to activate BAT is logical. In mice, pharmacological activation of this receptor drives brown adipocyte activation and a substantial degree of energy expenditure. Nevertheless, there are still some limitations to this approach as a tool to study the potential of BAT. First, both white and brown adipocytes express the β3-adrenergic receptor. It is thus difficult to formally ascribe the beneficial effects of mirabegron solely to its direct action on BAT. Moreover, β3-adrenergic receptor agonism may not maximally activate BAT thermogenesis in humans. The thermogenic potential of this tissue may thus be underestimated. In fact, human brown adipocytes appear to be more responsive to β2-adrenergic receptor agonism (16). Moreover, the natural response of BAT to cold exposure is not entirely mimicked by agonism of adipocyte β-adrenergic receptors. In fact, β-adrenergic receptor–independent mechanisms enhancing BAT thermogenesis exist (17). Cold induces a notable remodeling of adipose tissue, including changes in immune cell composition and vascular remodeling (18). Immune cell–derived signals, in turn, amplify the effects of catecholamines on thermogenic gene activation. As such, the identification of new strategies to better mimic the wide-ranging effects of cold exposure on brown fat tissue — not just mature adipocytes — may enable investigators to test the true potential of this tissue.

## We agree on a need for more BAT

Both Carpentier and Cypess agree on one important issue. If the abundance of functional thermogenic fat tissue can be increased in humans, then the chance of a therapeutic benefit is more likely. Since 2008, several advances have been made in our understanding of the developmental origins of brown adipocytes and the identity of their progenitor cells in adulthood. Moreover, animal models demonstrate the clear potential to reprogram mature, white adipocytes into a thermogenic brown- and beige-like cell through targeting of “transcriptional brakes” on the thermogenic gene program (19). The natural capacity of white adipocytes to activate a thermogenic phenotype is highlighted by the browning of WAT depots that occurs in humans suffering from severe burns or in those with pheochromocytomas (20, 21). Advancements in the technology supporting cell-based therapies and organoid development may make it possible to leverage our knowledge of adipocyte development for therapeutic benefit. Ultimately, successful translation of any approach to the clinic will depend on how putative BAT therapeutics stack up against current medicinal approaches. Emerging incretin-based therapies are proving to be effective for the treatment of obesity and diabetes (22); however, not all will benefit from these drugs for various reasons and there is room for complementary approaches. For example, one can envision leveraging adipose thermogenesis as means to help maintain weight loss.

## Concluding Remarks

Our understanding of BAT biology continues to increase at an exciting and rapid pace. The physiological importance of BAT in humans remains unclear, as is whether BAT dysfunction is part of the underlying problem leading to aspects of cardiometabolic disease. Nevertheless, adipose thermogenesis does not have to be part of the problem for it to be part of the solution. Patience and careful and rigorous science will be needed to fully understand if and how the emerging concepts in the field can be leveraged for the development of therapeutic strategies.

## Version 1 12/01/2023

Electronic publication
